# Subgroup-Specific Osteoporosis Risk in Chronic Kidney Disease: Insights from a Nationwide Korean Cohort

**DOI:** 10.3390/biomedicines13081956

**Published:** 2025-08-11

**Authors:** Ho Suk Kang, Joo-Hee Kim, Woo Jin Bang, Dae Myoung Yoo, Kyeong Min Han, Nan Young Kim, Hyo Geun Choi, Ha Young Park, Mi Jung Kwon

**Affiliations:** 1 Division of Gastroenterology, Department of Internal Medicine, Hallym University Sacred Heart Hospital, Hallym University College of Medicine, Anyang 14068, Republic of Korea; hskang76@hallym.or.kr; 2 Division of Pulmonary, Allergy, and Critical Care Medicine, Department of Medicine, Hallym University Sacred Heart Hospital, Hallym University College of Medicine, Anyang 14068, Republic of Korea; luxjhee@gmail.com; 3 Department of Urology, Hallym University Sacred Heart Hospital, Hallym University College of Medicine, Anyang 14068, Republic of Korea; yybbang@hallym.or.kr; 4 Hallym Data Science Laboratory, Hallym University College of Medicine, Anyang 14068, Republic of Korea; ydm1285@naver.com (D.M.Y.); km_han@hallym.ac.kr (K.M.H.); 5 Hallym Institute of Translational Genomics and Bioinformatics, Hallym University Medical Center, Anyang 14068, Republic of Korea; honeyny78@gmail.com; 6 Suseo Seoul E.N.T. Clinic, 10, Bamgogae-ro 1-gil, Gangnam-gu, Seoul 06349, Republic of Korea; mdanalytics@naver.com; 7 Department of Pathology, Busan Paik Hospital, Inje University College of Medicine, Busan 47392, Republic of Korea; hy08.park@gmail.com; 8 Department of Pathology, Hallym University Sacred Heart Hospital, Hallym University College of Medicine, Anyang 14068, Republic of Korea

**Keywords:** chronic kidney disease, osteoporosis, longitudinal follow-up study, nationwide health insurance research database, propensity score overlap weighting, risk assessment

## Abstract

**Background/Objectives**: Chronic kidney disease (CKD) and osteoporosis are critical public health concerns, particularly among older adults, due to their prevalence and associated complications. While CKD-related disruptions in bone mineral metabolism are believed to increase osteoporosis risk, this relationship remains unclear in diverse populations such as Korea. **Methods**: This longitudinal cohort study utilized data from the Korean National Health Insurance Service Health Screening Cohort (2002–2019), including 13,622 patients with newly diagnosed CKD and 54,488 matched controls. CKD was defined as having at least two outpatient or inpatient records with ICD-10 codes N18 or N19 and/or evidence of dialysis treatment claims, following a 1-year washout period to exclude prevalent cases. Individuals with a prior history of osteoporosis or incomplete baseline data were excluded. Propensity score overlap weighting was applied, and adjusted hazard ratios (HRs) with 95% confidence intervals (CIs) were calculated using Cox proportional hazards models, with subgroup analyses based on demographic and clinical factors. **Results**: CKD was not associated with an increased risk of osteoporosis. On the contrary, CKD patients exhibited an 18% lower risk of developing osteoporosis compared to controls (HR = 0.82, 95% CI: 0.77–0.87, *p* < 0.001). This inverse association was more pronounced among women, non-smokers, individuals with low alcohol consumption, and those with a higher comorbidity burden. **Conclusions**: These findings suggest that certain subgroups of CKD patients may have a reduced risk of osteoporosis, highlighting the importance of individualized risk assessment and tailored preventive strategies.

## 1. Introduction

Chronic kidney disease (CKD) and osteoporosis are major global health concerns, particularly among older adults, as both conditions are associated with severe complications and significant public health burdens [1,2,3]. Osteoporosis, characterized by reduced bone density and increased fracture risk, affects over 200 million people worldwide, resulting in substantial healthcare costs [4]. In Korea, 22.4% of individuals aged 50 years and older are diagnosed with osteoporosis, with prevalence rates significantly higher in women (35.5%) compared to men (7.5%) [5,6]. CKD is a progressive condition affecting over 10% of the global population, totaling more than 800 million people [2]. In Korea, the prevalence is similar, with 10–13% of the population impacted [1]. This underscores CKD’s significant public health burden worldwide and within Korea. These conditions often overlap [7,8], with CKD-related complications such as disrupted bone mineral metabolism contributing to an increased risk of osteoporosis [9,10].

Both CKD and osteoporosis share common risk factors, including aging, lifestyle behaviors, and socioeconomic status [11,12]. While previous studies have suggested that CKD—particularly in its advanced stages—may contribute to bone mineral density loss through mechanisms such as calcium–phosphorus imbalance, systemic inflammation, and secondary hyperparathyroidism [8,9], the overall relationship between CKD and osteoporosis remains poorly understood. Despite biologically plausible connections, epidemiological evidence is inconsistent. Some studies report an increased risk of osteoporosis and fractures among CKD patients, especially in advanced stages [9,10], whereas others find no significant association or even paradoxical protective trends in certain subgroups [11,13]. These discrepancies may arise from variations in study design, population characteristics, CKD definitions, or confounder adjustment [12,14,15]. Additionally, most available evidence originates from Western populations [8,16], limiting its applicability to Asian populations.

Korea’s rapidly aging population, coupled with the rising prevalence of chronic diseases [17], underscores the need to better understand the intersection of CKD and osteoporosis within this unique demographic. Although healthcare access and health behaviors have improved in recent decades, large-scale, population-based studies exploring this association in Korea are lacking. This gap is particularly concerning given the high prevalence of both CKD and osteoporosis in the Korean population [1,6]. Therefore, research using intensive methodology and nationally representative data is essential to clarify this relationship and inform effective prevention strategies for high-risk groups.

To address this gap, this study evaluates the association between CKD and osteoporosis risk in the Korean population, which may vary based on individual patient characteristics, such as age, sex, socioeconomic status, and comorbid conditions. We examined whether certain lifestyle or demographic factors (e.g., age, sex, smoking, rural residence) might modify this risk within the CKD population. To fulfill this, we conducted a long-term observational study using data from the Korean national public healthcare system, analyzing the association between CKD and osteoporosis risk while accounting for potential confounding factors. By leveraging nationwide, longitudinal data and robust analytical techniques, this research could help to inform more targeted prevention strategies and improve clinical outcomes for CKD patients, addressing an important public health concern.

## 2. Materials and Methods

### 2.1. Research Design, Data Resource, and Cohort Selection

This study analyzed data from the Health Screening Cohort of the Korean National Health Insurance Service, a comprehensive dataset that provides sociodemographic and clinical information on a representative sample of Korean adults, encompassing the period from 2002 to 2019. Participants aged 40–79 years who underwent health screenings during 2002–2003 and were followed until 2019 [18]. The dataset comprises 514,866 participants, selected through 10% simple random sampling from all eligible individuals during the enrollment period [19]. The database is fully anonymized by the government and employs International Classification of Diseases, 10th Revision (ICD-10) codes for healthcare information and standardized disease diagnosis. This study was approved by the Ethics Committee of Hallym University (IRB No. 2019-10-023), with a waiver of written informed consent due to the use of anonymized secondary data. All analyses were conducted in compliance with the guidelines and regulations set forth by the Ethics Committee of Hallym University.

This retrospective cohort study evaluated the impact of chronic kidney disease (CKD) on osteoporosis incidence using a large-scale dataset from the Korean National Health Insurance Service, which included 514,866 individuals aged ≥40 years and 895,300,177 medical claims recorded between 2002 and 2019. CKD was defined as having at least two outpatient or inpatient records with ICD-10 codes N18 (chronic kidney disease) or N19 (unspecified kidney failure), and/or receipt of dialysis based on Korean insurance claim codes (O7010, O7020, or O7070) [20]. To improve diagnostic accuracy and exclude prevalent cases, a washout period was applied in 2002, and only newly diagnosed cases from 2003 onward were included. Participants with a single CKD diagnosis, incomplete baseline data (e.g., missing BMI, blood pressure, or fasting glucose), or a prior history of osteoporosis were excluded to minimize misclassification and reverse causation.

For the control group, 497,388 eligible individuals without CKD-related diagnoses were initially identified. We first performed 1:4 exact matching based on age, sex, income level, and region of residence. The index date for each CKD patient—defined as the date of diagnosis—was assigned to a randomly selected matched control. Controls who had died or developed osteoporosis before the assigned index date were excluded, resulting in the removal of 442,340 individuals. After this matching step, we estimated propensity scores using the full set of baseline covariates and applied overlap weighting to further adjust for residual confounding and achieve covariate balance. The final study population included 13,622 CKD patients and 54,488 matched controls.

**Figure 1:**
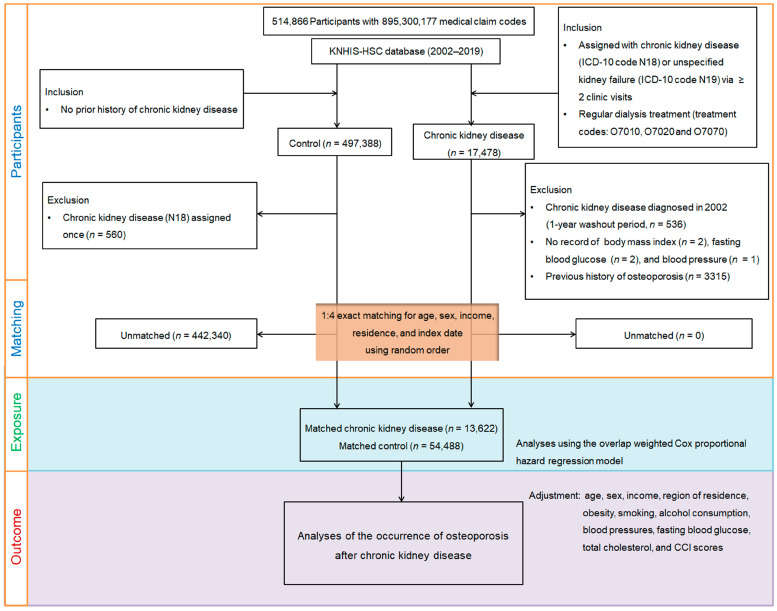
Flowchart of participant selection, 1:4 exact matching, and overlap weighting. Among 514,866 individuals aged ≥40 years, 13,622 patients with newly diagnosed CKD were identified based on ≥2 claims with ICD-10 codes N18/N19 and/or dialysis. After excluding those with prior osteoporosis, incomplete baseline data, or a single CKD record, 1:4 exact matching was performed by age, sex, income, and region. Each CKD patient’s index date (diagnosis date) was assigned to a randomly selected matched control. Controls with prior osteoporosis or death before the index date were excluded. Propensity score overlap weighting was applied to balance covariates between 13,622 CKD patients and 54,488 matched controls. Participants were followed for incident osteoporosis through 31 December 2019.

Osteoporosis was defined using ICD-10 codes M80 (with pathological fracture), M81 (without pathological fracture), and M82 (associated with other diseases) [21,22]. To ensure diagnostic accuracy, only individuals with at least two clinical visits and corresponding bone density assessments by X-ray or computed tomography (claim codes HC341–HC345, E7001–E7004) were considered as osteoporosis cases [21,22]. All participants were followed for osteoporosis incidence from the index date until 31 December 2019 (Figure 1).

### 2.2. Covariables

Participants were categorized by age (10 groups) and income level (5 tiers), with residential areas classified as urban or rural. Behavioral factors, including BMI, alcohol consumption, and smoking, were analyzed alongside physiological measures such as fasting glycemic values, blood pressure, and total cholesterol. The Charlson Comorbidity Index (CCI) was used to quantify overall health burden based on ICD-10 codes from each participant’s medical history, assigning scores ranging from 0 to 29 depending on the presence and severity of 17 comorbid conditions [23]. This index provides a standardized assessment of cumulative health impact [23]. The conditions included in the CCI are myocardial infarction, heart failure, peripheral vascular disease, cerebrovascular disease, dementia, chronic pulmonary disease, connective tissue disorders, peptic ulcer disease, liver disease, diabetes (with and without complications), paraplegia, renal disease, cancer, metastatic cancer, severe liver disease, and HIV/AIDS [23].

### 2.3. Statistical Analyses

To address confounding factors and ensure a balanced comparison between CKD patients and controls, this study utilized propensity score overlap weighting. This method was chosen for its ability to focus on the region of common support between groups, minimizing bias by excluding extreme outliers, maximizing the effective sample size by retaining all participants, and achieving balanced baseline characteristics across groups [24,25]. Unlike traditional propensity score matching, which discards unmatched participants and may introduce selection bias, overlap weighting creates a pseudo-population in which the baseline features of CKD and control groups are nearly identical, mimicking the properties of a randomized controlled trial [24,25].

Specifically, propensity scores were estimated through a logistic regression model that included multiple variables [26]. Overlap weights were then derived, assigning higher weights to participants with similar probabilities of being in either group, ensuring better comparability and accounting for complex relationships between covariates and group membership [24,25]. This ensures a more comprehensive adjustment for confounding factors. This approach allowed for a robust comparison of osteoporosis risk between CKD and control groups [25]. A standardized difference of less than 0.20 was considered indicative of a well-balanced dataset between the groups and minimized baseline differences [27].

Crude incidence rates and incidence rate differences were calculated as the number of events per 1000 person-years by dividing event counts by total person-years. Kaplan–Meier estimators were employed to evaluate osteoporosis incidence over time, while Cox proportional hazards regression models were used to estimate HRs and 95% CIs, with proportional hazards assumptions confirmed. Subgroup analyses were performed to examine variations by demographic and clinical factors. Statistical analyses were conducted using SAS 9.4 software (SAS Institute Inc., Cary, NC, USA), with significance set at *p*-values < 0.05 for two-tailed tests.

## 3. Results

### 3.1. Baseline Characteristics of the Participants

The initial traits of participants prior to and following propensity score overlap weighting are summarized (Table 1). Prior to adjustment, imbalances were detected between the CKD group and the control group in terms of systolic blood pressure, fasting glycemic values, and CCI scores. Subjects in the CKD group were less likely to exhibit a normal weight and more likely to have higher systolic blood pressure, fasting glycemic values, and CCI scores compared to the controls.

After overlap propensity score weighting, standardized differences for all variables were reduced to ≤0.20, achieving an equal distribution of traits between the CKD and control groups. The standardized differences for weight status categories, systolic blood pressure, fasting glycemic values, and CCI scores were reduced to 0.00, with similar reductions observed for other covariates, indicating effective adjustment for confounding variables.

### 3.2. Association Between CKD and Osteoporosis Likelihood

**Figure 2:**
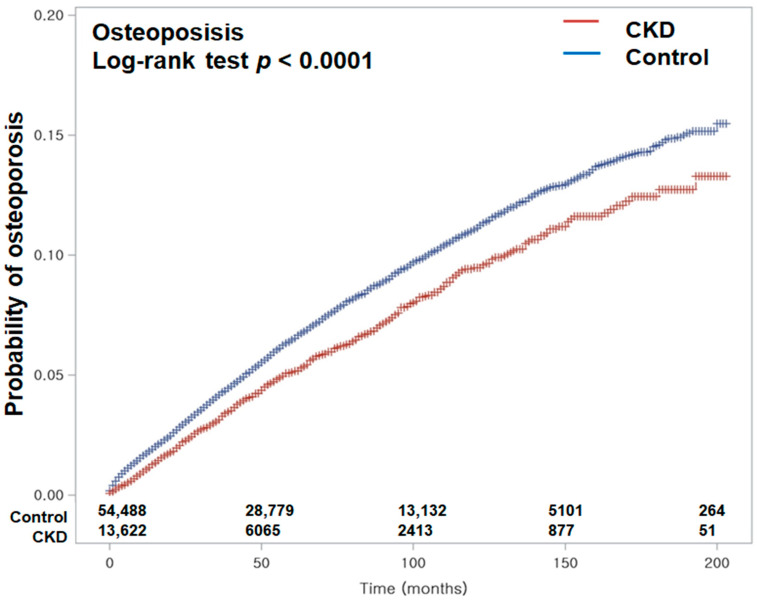
Kaplan–Meier curves depicting the cumulative incidence of osteoporosis in patients with chronic kidney disease (CKD) and in control subjects over a 16-year period from the index date. The Kaplan–Meier analysis with the log-rank test demonstrated a significantly lower incidence of osteoporosis in the CKD group compared to the control group during the 16-year follow-up period.

The incidence rates of osteoporosis in the CKD and control counterpart groups were 11.10 and 13.40 per 1000 person-years, respectively (difference in incidence rates: HR −2.30, 95% CI: −3.39–−1.33). After overlap-weighting adjustment for all demographics and medical comorbidities, Cox regression analysis disclosed that CKD participants exhibited a lower incidence of osteoporosis (HR = 0.82, 95% CI: 0.77–0.87, *p* < 0.001) (Table 2). The Kaplan–Meier analysis with log-rank test displayed a more reduced chance of osteoporosis in patients suffering CKD than in those without CKD during a 16-year period commencing from the index date (*p* < 0.0001; Figure 2).

### 3.3. Subgroup Analyses

Subgroup analyses (Table 3) revealed that the reduced risk of osteoporosis associated with CKD was consistent across various demographic and clinical factors. Female CKD patients exhibited a markedly reduced risk of developing osteoporosis (HR = 0.75, 95% CI: 0.69–0.82, *p* < 0.001), indicating a protective association. Conversely, in male CKD patients, the association was not substantial (HR = 0.95, 95% CI: 0.86–1.05, *p* = 0.348).

Non-smokers with CKD demonstrated a substantially lower likelihood of osteoporosis (HR = 0.78, 95% CI: 0.72–0.84, *p* < 0.001) compared to smokers, where the association lacked statistical significance (HR = 1.00, 95% CI: 0.87–1.15, *p* = 0.956). Similarly, CKD patients who consumed alcohol minimally (less than once per week) showed a greater protective effect (HR = 0.79, 95% CI: 0.73–0.85, *p* < 0.001) relative to those who consumed alcohol more frequently (HR = 0.99, 95% CI: 0.86–1.15, *p* = 0.898).

CKD individuals with higher comorbidity scores (CCI ≥ 1) exhibited a more pronounced reduction in osteoporosis risk, whereas patients with low comorbidity burdens showed no association (HR = 0.92, 95% CI: 0.84–1.02, *p* = 0.103).

Variations were also observed across age groups, income levels, and rural versus urban residency, weight status, total cholesterol levels, blood pressure, fasting glycemic values, or generally demonstrating a slightly protective effect.

## 4. Discussion

This longitudinal cohort study, utilizing nationwide large-scale data, demonstrated no notable rise in the overall occurrence of osteoporosis among CKD patients compared to controls throughout the 16-year observation period. Notably, CKD patients demonstrated an 18% reduction in the likelihood of developing osteoporosis (95% CI = 0.77–0.87), with this effect being particularly pronounced among women, non-smokers, individuals with minimal alcohol intake, and those with a high comorbidity burden. These findings highlight the complex interplay between CKD and osteoporosis, suggesting potential protective factors in CKD patients that may lower osteoporosis risk. They emphasize the importance of subgroup-specific analyses and the consideration of demographic and clinical characteristics when evaluating osteoporosis risk in the Korean population aged over 40.

Previous studies have identified CKD as a contributing factor to bone mineral density loss due to disrupted calcium–phosphorus metabolism, systemic inflammation, and secondary hyperparathyroidism [9]. However, our findings indicated a more nuanced relationship between CKD and osteoporosis risk. Our results appear to contrast with prior reviews reporting an increased osteoporosis risk in CKD, largely driven by advanced stages and dialysis populations [11,28]. This discrepancy may reflect differences in study populations, disease severity, methodologies, and healthcare systems [12,14,15]. Notably, many earlier studies focused on patients with late-stage CKD, where disturbances in mineral metabolism are more pronounced [9,10,11,28]. In contrast, our study included individuals across all CKD stages, likely capturing a broader clinical spectrum, including early-stage patients with milder biochemical abnormalities. Additionally, the reduced osteoporosis risk identified within our study population could be partially attributed to the widespread implementation of early medical interventions and nationwide osteoporosis screening programs in Korea, which promote early detection and management under the National Health Insurance Service [5,6]. These public health efforts may be particularly effective in mitigating osteoporosis risk in specific subgroups. Taken together, our findings suggest that the association between CKD and osteoporosis is not uniform but rather may vary depending on demographic, clinical, and healthcare-related factors. By incorporating comorbidities, socioeconomic status, and lifestyle characteristics into an overlap-weighted Cox regression model, our study showed inverse estimates of the relationship between CKD and osteoporosis risk in certain subgroups. The use of propensity score overlap weighting may strengthen causal inference by minimizing confounding and improving covariate balance, offering a more reliable assessment of this complex association [25].

In the present study, subgroup analyses revealed that demographic, lifestyle, and clinical factors significantly influenced osteoporosis risk among patients with CKD. A more pronounced inverse association between CKD and osteoporosis was observed in females, non-smokers, individuals with minimal alcohol consumption, and those with a higher comorbidity burden. While reduced exposure to conventional osteoporosis risk factors likely contributes to these findings [11], additional biological mechanisms may also be involved. Healthy lifestyle behaviors, including smoking cessation and low alcohol intake, may lower systemic inflammation and oxidative stress—key drivers of bone loss—and are often associated with greater engagement in preventive healthcare [11]. In addition, sex- and hormone-related differences may underlie the stronger protective effect observed in women [12]. Estrogen has well-documented bone-preserving effects, and in early CKD stages, some degree of estrogenic activity may be preserved, potentially helping delaying bone loss [12,14,29]. This preservation of estrogenic activity can act as a protective factor against the bone loss that is commonly seen in later stages of CKD and in conditions like osteoporosis [12,29]. Although other risk modifiers include older age, postmenopausal status, low body mass index, and comorbidities such as vascular or chronic inflammatory diseases [10,11,30,31], women with CKD may also experience unique hormonal adaptations that influence bone turnover differently than in men [29]. In addition, CKD patients with comorbidities often receive regular monitoring and treatment for mineral and bone disorders, including vitamin D supplementation, phosphate binders, and parathyroid hormone modulators [13,32]. These interventions may mitigate bone deterioration and lower the likelihood of osteoporosis [12,32]. In dialysis patients, targeted treatments such as parathyroidectomy have also demonstrated protective effects, supporting the importance of individualized management strategies in modifying osteoporosis risk [30].

Genetic factors also contribute significantly to osteoporosis susceptibility, particularly among Korean women [33]. Using whole-genome comparative expression profiling, gene expression analysis, and association studies, a significant relationship was identified between single-nucleotide polymorphisms in five genes—*ANXA6*, *COL5A1*, *ENO1*, *MYOF*, and *SCARA5*—and bone mineral density and/or osteoporosis in a cohort of 3570 Korean women [33]. In addition, the *Apa*I polymorphism in the vitamin D receptor gene—associated with bone mineral density and fracture risk in Korean postmenopausal women—may influence individual responsiveness to vitamin D [34]. Together, these findings may suggest the multifactorial nature of osteoporosis risk in CKD, involving genetic predisposition, behavioral, hormonal, and metabolic pathways. Subgroup-specific differences should be considered when developing targeted prevention strategies in clinical practice.

This study leverages representative, nationwide data from the Korean National Health Insurance Service Health Screening Cohort, offering an in-depth analysis that accounts for economic background, behavioral risk factors, and concurrent health conditions. By incorporating complete medical histories from healthcare facilities nationwide, the findings are both highly precise and broadly generalizable. To the best of our knowledge, this is the most extensive nationwide follow-up analysis examining the association between osteoporosis and CKD risk in Korean adults. The study’s robustness is further bolstered by the use of overlap-weighted propensity score matching, which minimizes selection bias and enables subgroup analyses comparable to randomized clinical trials [25]. With a 16-year follow-up period, this long-term observational study offers important perspectives on the relationship between CKD and osteoporosis, with adjustments for potential confounders further enhancing the credibility and applicability of the findings [7].

This study has several limitations that should be considered. First, its observational design precludes causal inference, and residual confounding may persist despite robust statistical adjustments using exact matching and propensity score overlap weighting. Second, the use of ICD-10 codes and claims data may introduce misclassification bias, as undiagnosed or inaccurately coded cases of CKD and osteoporosis could affect classification. Third, the lack of detailed clinical data, such as estimated glomerular filtration rate or albuminuria, prevented stratification by CKD severity and limited our ability to assess stage-specific associations with osteoporosis risk. Although dialysis claim codes enabled the identification of end-stage renal disease, earlier CKD stages could not be distinguished. Fourth, healthy user bias may have influenced our findings. CKD patients often engage more frequently with the healthcare system, potentially leading to earlier detection and better management of osteoporosis, which could partially explain the lower observed risk in this group. Fifth, although we discussed potential effects of medications such as vitamin D, phosphate binders, and parathyroid hormone modulators, detailed information on medication use—including dosage, frequency, and adherence—was not available in the Korean National Health Insurance Service dataset. Thus, the role of pharmacologic treatment in modifying osteoporosis risk could not be directly assessed. Sixth, genetic factors, such as vitamin D receptor gene polymorphisms, which may influence bone metabolism and treatment response, were not included due to the absence of genomic data. Seventh, treatment protocols for CKD and osteoporosis in Korea, including standardized national screening and early intervention programs, may differ from those in other countries. These system-level healthcare differences could affect treatment patterns and osteoporosis detection rates, potentially limiting the generalizability of our findings across international settings. Lastly, our study population consisted of Korean adults aged ≥40 years, which may restrict generalizability to younger individuals or populations with different ethnic or geographic backgrounds.

## 5. Conclusions

In conclusion, this nationwide study suggests that overall, CKD patients in Korea may not experience a heightened risk of osteoporosis compared to individuals without CKD and even exhibit a slightly lower likelihood, particularly among females, non-smokers, those with minimal alcohol consumption, and individuals with a high comorbidity burden. These findings emphasize the need for individualized risk assessments and targeted prevention strategies, particularly for CKD patients with minimal lifestyle risk factors. Health professionals need to focus on early screening and tailored interventions to optimize bone health in this population. By identifying subgroup-specific variations in osteoporosis risk, this study may provide valuable insights for guiding targeted prevention strategies and improving clinical outcomes, potentially contributing to better public health policies in Korea.

## Figures and Tables

**Table 1:** Baseline characteristics of participants before and after propensity score overlap weighting.

Characteristics		Before Overlap-Weighting Adjustment			After Overlap-Weighting Adjustment		
		CKD	Control	Standardized Difference	CKD	Control	Standardized Difference
Age (y), *n* (%)				0.00			0.00
	40–44	96 (0.70)	384 (0.70)		73 (0.72)	73 (0.72)	
	45–49	349 (2.56)	1396 (2.56)		251 (2.49)	251 (2.49)	
	50–54	911 (6.69)	3644 (6.69)		654 (6.51)	654 (6.51)	
	55–59	1748 (12.83)	6992 (12.83)		1275 (12.69)	1275 (12.69)	
	60–64	2077 (15.25)	8308 (15.25)		1511 (15.04)	1511 (15.04)	
	65–69	2247 (16.50)	8988 (16.50)		1641 (16.33)	1641 (16.33)	
	70–74	2347 (17.23)	9388 (17.23)		1745 (17.37)	1745 (17.37)	
	75–79	2035 (14.94)	8140 (14.94)		1529 (15.22)	1529 (15.22)	
	80–84	1246 (9.15)	4984 (9.15)		935 (9.31)	935 (9.31)	
	≥85	566 (4.16)	2264 (4.16)		434 (4.32)	434 (4.32)	
Sex, *n* (%)				0.00			0.00
	Male	10,422 (76.51)	41,688 (76.51)		7718 (76.81)	7718 (76.81)	
	Female	3200 (23.49)	12,800 (23.49)		2331 (23.19)	2331 (23.19)	
Income, *n* (%)				0.00			0.00
	1 (lowest)	2292 (16.83)	9168 (16.83)		1679 (16.71)	1679 (16.71)	
	2	1625 (11.93)	6500 (11.93)		1201 (11.95)	1201 (11.95)	
	3	1958 (14.37)	7832 (14.37)		1435 (14.28)	1435 (14.28)	
	4	2772 (20.35)	11,088 (20.35)		2035 (20.26)	2035 (20.26)	
	5 (highest)	4975 (36.52)	19,900 (36.52)		3698 (36.80)	3698 (36.80)	
Region of residence, *n* (%)				0.00			0.00
	Urban	5899 (43.30)	23,596 (43.30)		4351 (43.30)	4351 (43.30)	
	Rural	7723 (56.70)	30,892 (56.70)		5697 (56.70)	5697 (56.70)	
Obesity ^†^, *n* (%)				0.15			0.00
	Underweight	320 (2.35)	1645 (3.02)		246 (2.45)	246 (2.45)	
	Normal	4128 (30.30)	19,007 (34.88)		3131 (31.16)	3131 (31.16)	
	Overweight	3638 (26.71)	15,191 (27.88)		2721 (27.08)	2721 (27.08)	
	Obese I	4958 (36.40)	17,126 (31.43)		3565 (35.48)	3565 (35.48)	
	Obese II	578 (4.24)	1519 (2.79)		386 (3.84)	386 (3.84)	
Smoking status, *n* (%)				0.03			0.00
	Non-smoker	7966 (58.48)	32,210 (59.11)		5884 (58.56)	5884 (58.56)	
	Past smoker	1628 (11.95)	6776 (12.44)		1228 (12.22)	1228 (12.22)	
	Current smoker	4028 (29.57)	15,502 (28.45)		2936 (29.22)	2936 (29.22)	
Alcohol consumption, *n* (%)				0.07			0.00
	<1 time a week	9355 (68.68)	35,539 (65.22)		6803 (67.71)	6803 (67.71)	
	≥1 time a week	4267 (31.32)	18,949 (34.78)		3245 (32.29)	3245 (32.29)	
SBP, mean (SD ^‡^)		131.92 (18.53)	128.52 (16.21)	0.20	130.88 (15.44)	130.88 (7.30)	0.00
DBP, mean (SD ^‡^)		79.06 (11.65)	78.24 (10.35)	0.07	78.80 (9.87)	78.80 (4.55)	0.00
Fasting blood glucose, mean (SD ^‡^)		117.15 (51.35)	104.18 (28.75)	0.31	110.84 (33.49)	110.84 (17.33)	0.00
Total cholesterol, mean (SD ^‡^)		189.90 (45.75)	192.44 (38.27)	0.06	190.09 (38.90)	190.09 (16.60)	0.00
CCI score, mean (SD ^‡^)		2.18 (2.22)	1.09 (1.73)	0.55	1.82 (1.68)	1.82 (0.99)	0.00
Osteoporosis, *n* (%)		632 (4.64)	3695 (6.78)	0.09	476 (4.74)	685 (6.82)	0.09

Abbreviations: CCI, Charlson Comorbidity Index; SBP, systolic blood pressure; DBP, diastolic blood pressure; CKD, chronic kidney disease; SD, standard deviation. ^†^ Obesity (body mass index, kg/m^2^) was categorized as underweight (<18.5), normal (18.5–23), overweight (23–25), obese I (25–30), and obese II (≥30). ^‡^ After applying overlap weighting, standard deviations—particularly in the control group—decreased as expected due to the weighting mechanism. Overlap weighting emphasizes individuals with intermediate propensity scores (i.e., closer to 0.5), thereby down-weighting those with extreme scores and reducing variance within covariates.

**Table 2:** Crude and propensity score overlap-weighted HRs and 95% CIs of CKD for osteoporosis, with subgroup analyses according to age, sex, income, and region of residence.

		N of Event/ N of Total (%)	Follow-Up Duration (PY)	IR per 1000 (PY)	HRs for Osteoporosis	*p*
					Overlap-Weighted Model ^†^	
Total participants						
	CKD	632/13,622 (4.64)	57,063	11.10	0.82 (0.77–0.87)	**<0.001**
	Control	3695/54,488 (6.78)	275,039	13.40	1	
Aged < 70 years						
	CKD	394/7428 (5.30)	39,274	10.00	0.87 (0.80–0.95)	**0.001**
	Control	2119/29,712 (7.13)	184,839	11.50	1	
Aged ≥ 70 years						
	CKD	238/6194 (3.84)	17,789	13.40	0.74 (0.66–0.81)	**<0.001**
	Control	1576/24,776 (6.36)	90,200	17.50	1	
Male						
	CKD	260/10,422 (2.49)	43,098	6.03	0.95 (0.86–1.05)	0.348
	Control	1312/41,688 (3.15)	209,382	6.27	1	
Female						
	CKD	372/3200 (11.63)	13,965	26.60	0.75 (0.69–0.82)	**<0.001**
	Control	2383/12,800 (18.62)	65,657	36.30	1	
Low-income group						
	CKD	289/5875 (4.92)	24,196	11.90	0.82 (0.75–0.90)	**<0.001**
	Control	1712/23,500 (7.29)	118,892	14.40	1	
High-income group						
	CKD	343/7747 (4.43)	32,867	10.40	0.82 (0.75–0.89)	**<0.001**
	Control	1983/30,988 (6.40)	156,147	12.70	1	
Urban resident						
	CKD	260/5899 (4.41)	26,370	9.86	0.89 (0.80–0.98)	**0.02** **0**
	Control	1394/23,596 (5.91)	125,144	11.10	1	
Rural resident						
	CKD	372/7723 (4.82)	30,693	12.10	0.78 (0.71–0.84)	**<0.001**
	Control	2301/30,892 (7.45)	149,895	15.40	1	

Abbreviation: CKD, chronic kidney disease; IR, incidence rate; PY, person-year; HR, hazard ratio; CI, confidence interval. Bold indicates statistical significance at *p* < 0.05. ^†^ Adjusted for age, sex, income, region of residence, obesity, smoking, alcohol consumption, systolic blood pressure, diastolic blood pressure, fasting blood glucose, total cholesterol, and Charlson Comorbidity Index scores.

**Table 3:** Subgroup analyses of the crude and propensity score overlap-weighted hazard ratios and 95% confidence intervals of CKD for osteoporosis.

		N of Event/ N of Total (%)	Follow-Up Duration (PY)	IR per 1000 (PY)	HRs for Osteoporosis (95% CI)	*p*
					Overlap-Weighted Model ^†^	
Underweight						
	CKD	13/320 (4.06)	977	13.30	0.54 (0.37–0.80)	**0.002**
	Control	149/1645 (9.06)	6890	21.60	1	
Normal weight						
	CKD	220/4128 (5.33)	16,641	13.20	0.86 (0.78–0.96)	**0.006**
	Control	1400/19,007 (7.37)	95,134	14.70	1	
Overweight						
	CKD	154/3638 (4.23)	15,956	9.65	0.75 (0.66–0.85)	**<0.001**
	Control	979/15,191 (6.44)	78,425	12.50	1	
Obese						
	CKD	245/5536 (4.43)	23,489	10.40	0.87 (0.78–0.97)	**0.011**
	Control	1167/18,645 (6.26)	94,590	12.30	1	
Non-smoker						
	CKD	490/7966 (6.15)	34,611	14.20	0.78 (0.72–0.84)	**<0.001**
	Control	2991/32,210 (9.29)	163,995	18.20	1	
Past and current smoker						
	CKD	142/5656 (2.51)	22,452	6.32	1.00 (0.87–1.15)	0.956
	Control	704/22,278 (3.16)	111,044	6.34	1	
Alcohol consumption <1 time a week						
	CKD	515/9355 (5.51)	40,030	12.90	0.79 (0.73–0.85)	**<0.001**
	Control	3034/35,539 (8.54)	180,455	16.80	1	
Alcohol consumption ≥1 time a week						
	CKD	117/4267 (2.74)	17,033	6.87	0.99 (0.86–1.15)	0.898
	Control	661/18,949 (3.49)	94,584	6.99	1	
SBP < 140 mmHg and DBP < 90 mmHg						
	CKD	415/8874 (4.68)	35,490	11.70	0.85 (0.79–0.91)	**<0.001**
	Control	2625/39,858 (6.59)	196,123	13.40	1	
SBP ≥ 140 mmHg or DBP ≥ 90 mmHg						
	CKD	217/4748 (4.57)	21,573	10.10	0.76 (0.68–0.86)	**<0.001**
	Control	1070/14,630 (7.31)	78,916	13.60	1	
Fasting blood glucose < 100 mg/dL						
	CKD	355/6193 (5.73)	27,927	12.70	0.85 (0.78–0.92)	**<0.001**
	Control	2362/29,627 (7.97)	161,535	14.60	1	
Fasting blood glucose ≥ 100 mg/dL						
	CKD	277/7429 (3.73)	29,136	9.51	0.77 (0.70–0.86)	**<0.001**
	Control	1333/24,861 (5.36)	113,504	11.70	1	
Total cholesterol < 200 mg/dL						
	CKD	337/8467 (3.98)	33,103	10.20	0.79 (0.72–0.86)	**<0.001**
	Control	1965/32,334 (6.08)	156,177	12.60	1	
Total cholesterol ≥ 200 mg/dL						
	CKD	295/5155 (5.72)	23,960	12.30	0.87 (0.79–0.95)	**0.002**
	Control	1730/22,154 (7.81)	118,862	14.60	1	
CCI scores = 0						
	CKD	195/4092 (4.77)	18,807	10.40	0.92 (0.84–1.02)	0.103
	Control	1827/30,286 (6.03)	158,107	11.60	1	
CCI scores = 1						
	CKD	105/2221 (4.73)	8820	11.90	0.83 (0.72–0.97)	**0.016**
	Control	755/9633 (7.84)	48,522	15.60	1	
CCI scores ≥ 2						
	CKD	332/7309 (4.54)	29,436	11.30	0.71 (0.64–0.80)	**<0.001**
	Control	1113/14,569 (7.64)	68,410	16.30	1	

Abbreviation: CKD, chronic kidney disease; IR, incidence rate; PY, person-year; HR, hazard ratio; CI, confidence interval; CCI, Charlson Comorbidity Index. Bold indicates statistical significance at *p* < 0.05. ^†^ Adjusted for age, sex, income, region of residence, obesity, smoking, alcohol consumption, systolic blood pressure, diastolic blood pressure, fasting blood glucose, total cholesterol, and Charlson Comorbidity Index scores.

## Data Availability

All data are available from the database of National Health Insurance Sharing Service (NHISS) https://nhiss.nhis.or.kr/ (accessed on 1 October 2024). NHISS allows access to all of this data for any researcher who promises to follow the research ethics at some processing charge. If you want to access the data of this article, you can download it from the website after promising to follow the research ethics.
